# Implementation of Telerehabilitation Interventions for the Self-Management of Cardiovascular Disease: Systematic Review

**DOI:** 10.2196/17957

**Published:** 2020-11-27

**Authors:** Narayan Subedi, Jonathan C Rawstorn, Lan Gao, Harriet Koorts, Ralph Maddison

**Affiliations:** 1 School of Exercise and Nutrition Sciences Faculty of Health Deakin University Melbourne Australia; 2 School of Health and Social Development Faculty of Health Deakin University Melbourne Australia

**Keywords:** heart diseases, cardiac rehabilitation, telerehabilitation, implementation science, smartphone, systematic review

## Abstract

**Background:**

Coronary heart disease (CHD) is a leading cause of disability and deaths worldwide. Secondary prevention, including cardiac rehabilitation (CR), is crucial to improve risk factors and to reduce disease burden and disability. Accessibility barriers contribute to underutilization of traditional center-based CR programs; therefore, alternative delivery models, including cardiac telerehabilitation (ie, delivery via mobile, smartphone, and/or web-based apps), have been tested. Experimental studies have shown cardiac telerehabilitation to be effective and cost-effective, but there is inadequate evidence about how to translate this research into routine clinical practice.

**Objective:**

This systematic review aimed to synthesize research evaluating the effectiveness of implementing cardiac telerehabilitation interventions at scale in routine clinical practice, including factors underlying successful implementation processes, and experimental research evaluating implementation-related outcomes.

**Methods:**

MEDLINE, Embase, PsycINFO, and Global Health databases were searched from 1990 through November 9, 2018, for studies evaluating the implementation of telerehabilitation for the self-management of CHD. Reference lists of included studies and relevant systematic reviews were hand searched to identify additional studies. Implementation outcomes of interest included acceptability, appropriateness, adoption, feasibility, fidelity, implementation cost, penetration, and sustainability. A narrative synthesis of results was carried out.

**Results:**

No included studies evaluated the implementation of cardiac telerehabilitation in routine clinical practice. A total of 10 studies of 2250 participants evaluated implementation outcomes, including acceptability (8/10, 80%), appropriateness (9/10, 90%), adoption (6/10, 60%), feasibility (6/10, 60%), fidelity (7/10, 70%), and implementation cost (4/10, 40%), predominantly from the participant perspective. Cardiac telerehabilitation interventions had high acceptance among the majority of participants, but technical challenges such as reliable broadband internet connectivity can impact acceptability and feasibility. Many participants considered telerehabilitation to be an appropriate alternative CR delivery model, as it was convenient, flexible, and easy to access. Participants valued interactive intervention components, such as real-time exercise monitoring and feedback as well as individualized support. The penetration and sustainability of cardiac telerehabilitation, as well as the perspectives of CR practitioners and health care organizations, have received little attention in existing cardiac telerehabilitation research.

**Conclusions:**

Experimental trials suggest that participants perceive cardiac telerehabilitation to be an acceptable and appropriate approach to improve the reach and utilization of CR, but pragmatic implementation studies are needed to understand how interventions can be sustainably translated from research into clinical practice. Addressing this gap could help realize the potential impact of telerehabilitation on CR accessibility and participation as well as person-centered, health, and economic outcomes.

**Trial Registration:**

International Prospective Register of Systematic Reviews (PROSPERO) CRD42019124254; https://www.crd.york.ac.uk/prospero/display_record.php?RecordID=124254

## Introduction

Cardiovascular diseases (CVDs) are a leading cause of clinical (ie, death and disability), health, and economic burden globally, accounting for approximately 31% (17.9 million) of total deaths each year [1-3]. Coronary heart disease (CHD), including myocardial infarction (MI) and angina, is the most common and burdensome form of CVD [4,5]. CHD accounts for a high proportion of all CVD deaths and more disability-adjusted life years than diseases such as cancer and diabetes [4-6]. Therefore, secondary prevention interventions that support CVD management are critical to reducing disease burden and health care expenditure.

Cardiac rehabilitation (CR) is an essential component of secondary prevention for CHD that comprises coordinated, multifaceted interventions designed to improve physical, psychological, and social functioning [7-12]. CR includes medical evaluation, exercise prescription, cardiac risk factor modification, education, and counseling [13]. CR is safe, effective [14], and more cost-effective than no CR on overall health service expenditure [15-17]. Systematic reviews have shown that participation in center-based programs (ie, face-to-face delivery) reduces risks of hospital admissions and cardiac mortality, and improves health-related quality of life [14,18].

Despite these benefits, uptake and adherence of center-based CR are suboptimal [19-21]. Reasons for this are multifaceted [22-26], but accessibility-related factors, such as limited availability of programs, transportation, and parking, are prominent [22,24-28]. For these reasons, home-based delivery models have been tested to improve access and participation outside of clinical settings [29].

Home-based CR, which typically includes print resources, home visits, and/or telephone calls, has been shown to be as effective as center-based programs for improving health-related quality of life, CVD risk factors, and mortality [30]. However, few CR services offer home-based options (eg, less than one-quarter in the United Kingdom, United States, and Australia [30,31]). In addition, home-based programs are typically unable to provide the level of supervision, individualized coaching, and feedback from CR professionals that is common in center-based programs. Therefore, alternative delivery models that combine the accessibility of home-based programs with the comprehensive support of center-based CR are needed.

The use of information and communication technologies (ICTs) to connect participants and CR professionals, which is termed cardiac telerehabilitation [32], has been investigated as an alternative. Systematic reviews have demonstrated the effectiveness of cardiac telerehabilitation for improving cardiovascular risk factors and health-related quality of life [33-37]. However, early telerehabilitation interventions were mostly limited to telephone counseling, which limits the types of rehabilitation support that can be provided [38]. Technological innovations including mobile phones, particularly smartphones, and mobile broadband [39,40] have enabled more flexible cardiac telerehabilitation interventions [36,41-43].

Recent studies using cutting-edge technologies, such as smartphones, mobile apps, and the internet, have demonstrated that cardiac telerehabilitation can deliver more comprehensive services [44], including individualized real-time exercise monitoring and coaching, similar to center-based programs [45]. Growing evidence indicates telerehabilitation could substantially broaden the benefits and impact of CR; however, most interventions have only been evaluated in controlled experimental settings (eg, [41-45]). There is little evidence to guide the successful, scalable, sustainable translation of telerehabilitation into real-world settings [46-48]; that is, there is a lack of studies that have tested telerehabilitation interventions when delivered by health care staff in routine clinical practice.

Real-world implementation of an intervention is contextually dependent, influenced by individual (ie, personal characteristics), organizational (ie, hospital or service organization), community (ie, local government), and system-level (ie, government) factors, all of which are difficult to control in experimental designs [49]. Many public health interventions fail to be adopted or are less likely to be scaled and sustained when delivered in real-world settings, and the complexities and challenges involved in real-world implementation and scale-up are partly responsible for this lack of translational success [50]. A greater understanding of factors related to the implementation of interventions in practice settings is imperative for increasing population-level impact [51].

The purpose of this review was to synthesize research evaluating the implementation of cardiac telerehabilitation interventions when delivered in routine clinical practice.

## Methods

### Registration

This review was registered in PROSPERO (International Prospective Register of Systematic Reviews) (CRD42019124254) before screening search results, and was conducted according to the PRISMA (Preferred Reporting Items for Systematic Reviews and Meta-Analyses) statement [52,53].

### Information Sources and Search Strategy

Electronic databases—MEDLINE, Embase, PsycINFO, and Global Health—were searched between January 1990 and November 9, 2018, for studies that combined three concepts: telehealth, CVD, and implementation science. The search strategy was created for MEDLINE and modified for the other databases (see Multimedia Appendix 1).

### Eligibility and Study Selection

Eligible studies were those that evaluated the implementation of cardiac telerehabilitation in routine clinical practice or assessed implementation outcomes of interest in experimental studies, including randomized and nonrandomized designs, among adults (aged ≥18 years) with CHD (ie, MI, angina, and coronary revascularization).

Cardiac telerehabilitation interventions were defined as those with at least 50% of the program delivered via ICT, including any mobile phone (ie, feature phone or smartphone), web-based platforms, or wireless devices such as sensors. Implementation in routine clinical practice was defined as interventions delivered as part of existing CR services, without significant ongoing input from a research team.

Experimental studies were included as we anticipated few studies examining real-world implementation, and experimental studies provide the next best available evidence to advance the field; eligibility was not limited to randomized controlled trials (RCTs) to allow the inclusion of translational studies that used alternative study designs. In addition to other criteria, eligible experimental studies were those that assessed constructs defined in a taxonomy of key constructs related to the effective implementation of evidence-based interventions, including acceptability, adoption, appropriateness, feasibility, fidelity, implementation cost, penetration, and sustainability [54]. To acknowledge the importance of multiple stakeholder levels in successful implementation projects [55,56] and to meet the aims of this review, we assessed these constructs at the consumer (ie, participant), individual provider (ie, CR practitioner), and provider (ie, health care organization or institution) levels [54]. Constructs were defined as follows:

1. Acceptability: satisfaction among implementation stakeholders with different aspects of the intervention, such as content, delivery, and complexity. Stakeholders included health care consumers, practitioners, and health care organization operational staff who participated in, delivered, and oversaw the provision of CR services, respectively.
2. Adoption: rates of uptake or utilization of the intervention at the practitioner and/or health care organization level.
3. Appropriateness: program suitability or compatibility at the health care consumer, practitioner, and/or health care organization level.
4. Feasibility: practicability of the intervention for everyday use at the practitioner and/or health care organization level.
5. Fidelity: delivery of the intervention as designed.
6. Implementation cost: assessments of marginal cost, cost-effectiveness, or cost benefit.
7. Penetration: the degree to which the intervention was institutionalized within health care organizations.
8. Sustainability: continued delivery of the intervention beyond the study period, as well as characteristics of the implementation context that did or could influence the continuation of intervention delivery [54].

Feasibility and pilot studies were excluded from this review. To meet the aims of this review, it was important to include only interventions that had already undergone preliminary testing for feasibility and were considered by their respective authors as feasible for testing in the trial or delivery in practice. Conference abstracts, nonhuman studies, non-English-language papers, and grey literature were also excluded. Systematic reviews and study protocols were not eligible for inclusion; however, relevant systematic reviews were searched for eligible studies and cited where appropriate, and results articles were sought for relevant study protocols.

Search results were exported to a reference manager, EndNote X8 (Clarivate), for duplicate removal, then transferred to Rayyan (Qatar Computing Research Institute) for screening [57]. Records were assessed by NS, verified by JR and HK, and underwent full-text review if the title or abstract identified the specified population and intervention components.

### Data Extraction

Data describing eligibility, study design, participant and intervention characteristics, risk of bias, and outcomes of interest were extracted by NS using a standardized electronic form and verified by JR.

### Risk-of-Bias Assessment

Risks of selection, performance, detection, attrition, reporting, and other biases in included experimental studies were assessed using the Cochrane risk-of-bias tool [58]. Risks of bias in nonrandomized cohort studies were assessed using the Joanna Briggs Institute Critical Appraisal Checklist for Cohort Studies [59]. Risks of bias were assessed by NS and verified by JR. When available, risk-of-bias assessments were augmented with study protocols and clinical trial registrations.

### Data Synthesis

A narrative synthesis of the data was carried out in this review.

## Results

### Study Selection

**Figure 1:**
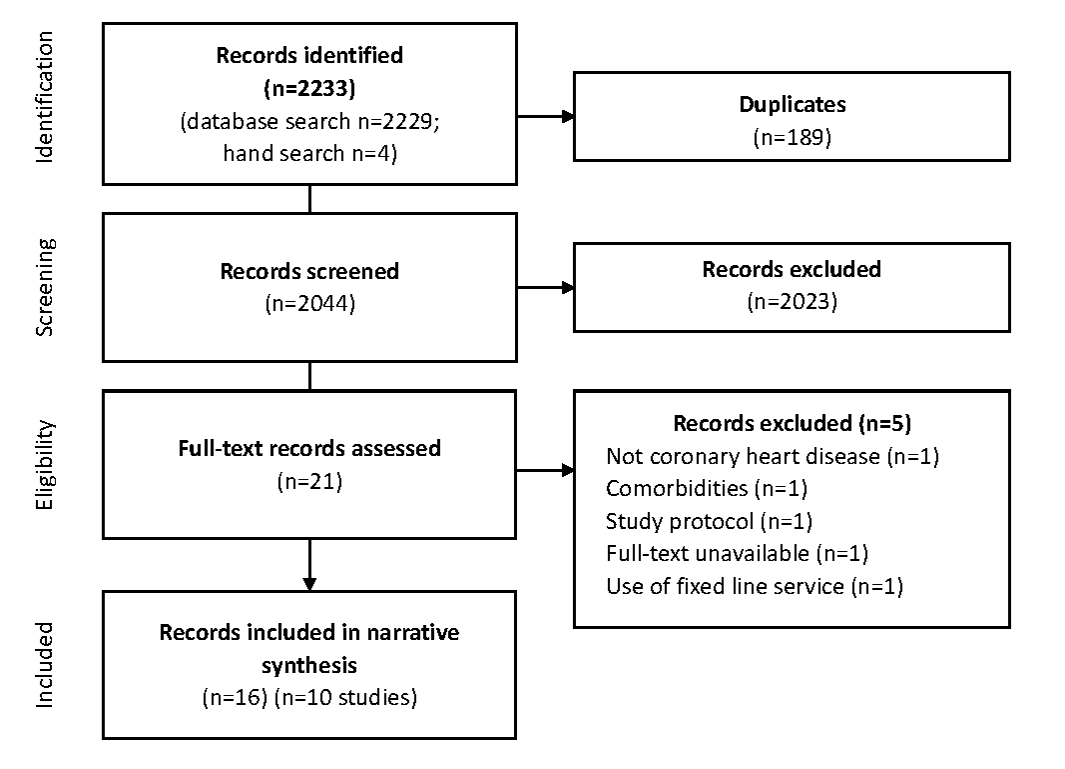
Summary of study selection process using the PRISMA (Preferred Reporting Items for Systematic Reviews and Meta-Analyses) flowchart.

In total, 2044 unique study reports were screened. From these, 21 underwent full-text review; 16 reports describing 10 studies (2250 participants in total) met the eligibility criteria and were included in the narrative synthesis [41-45,60-64]. Study selection is summarized in Figure 1.

### Characteristics of Included Studies

Included studies were published between 2013 and 2018; studies were conducted in developed countries, including New Zealand [42,43,45], Australia [41,44], Canada [61], the Netherlands [60], Poland [63], and the United States [62]. One multi-country study was carried out in Spain, Germany, and the United Kingdom [64].

No eligible studies were identified that evaluated the effectiveness of the implementation of cardiac telerehabilitation in routine clinical practice. Of the 10 included studies, 9 (90%) used randomized controlled experimental designs [41-45,60-62,64], while 1 (10%) used an uncontrolled pre-post intervention design [63].

The mean age of study participants ranged from 55 to 65 years, and most participants were male (72%-93%). All studies recruited participants via hospitals or community-based CR centers. Detailed characteristics of the included studies (ie, study design, treatments, and primary and implementation outcomes) are presented in Multimedia Appendix 2.

### Cardiac Telerehabilitation Intervention Characteristics

Out of 10 studies, 3 interventions (30%) were delivered using a mobile phone, smartphone, or web-based platform alone [41,61,62], while remaining interventions used combinations of web-based content, mobile phones or smartphones, and sensors. The most commonly targeted lifestyle risk factors were physical activity, diet, tobacco smoking, and medication adherence. Intervention duration ranged from 30 days [62] to 24 weeks [41-43] (see Multimedia Appendix 2).

Most interventions (6/10, 60%) comprised a messaging component (eg, SMS or push notifications) [41,43-45,61,62] to educate or motivate participants to improve self-management behaviors. Out of 10 interventions, 7 (70%) enabled communication between providers and participants via a web-based program, mobile phone or smartphone, and/or telephone [42,44,45,60,61,63,64]. Out of 10 interventions, 6 (60%) included exercise monitoring [42,44,45,60,63,64], including 2 (20%) that provided live guidance [64] or real-time monitoring and coaching during exercise [45]. Out of 10 studies, 5 (50%) delivered telerehabilitation in combination with usual care (ie, center- or community-based CR) [41-43,45,60], 4 (40%) delivered cardiac telerehabilitation alone [44,61,62,64], and 1 (10%) delivered a hybrid intervention comprising center-based and telerehabilitation components [63] (see Multimedia Appendix 2).

### Risk of Bias in the Included Studies

The quality of the included studies in the review varied (see Multimedia Appendix 2). Risk of bias was judged to be low in 6 out of 10 (60%) experimental studies [41-43,45,60,61] and high in 3 (30%) studies [44,62,64]. High risk of bias was judged due to incomplete outcome data and lack of blinding of participants and outcomes.

The single nonrandomized study (1/10, 10%) had a high risk of bias due to lack of a control group, not identifying confounding factors, and inadequate reporting of follow-up time [63].

### Implementation Outcomes

#### Overview

Included studies reported between three and six implementation outcomes. Appropriateness, acceptability, fidelity, adoption and feasibility, were assessed in 9 (90%), 8 (80%), 7 (70%), and 6 (60%), of the 10 studies, respectively; cost of intervention was assessed in only 4 (40%) studies, and penetration and sustainability were not assessed. Outcomes were predominantly assessed from a participant perspective, rather than from individual provider (ie, practitioner) or organizational perspectives. Implementation outcome findings are summarized below, with supporting data provided in Table 1 [41-45,60-71].

**Table 1:** Implementation outcomes for telerehabilitation interventions.

Study author, year, and implementation construct		Implementation outcomes
**Chow, 2015 [41,65]**		
	Acceptability	SMS intervention acceptability was 90.9% (279/307); request to stop SMS was 2.3% (7/307)
	Adoption	Focus groups reported high user engagement with saving and sharing SMS messages, receiving support from providers and family, and message personalization
	Appropriateness	SMS was useful: 90.9% (279/307) SMS was easy to understand: 96.7% (297/307) SMS was motivating for change: 77.2% (237/307); especially for diet (249/307, 81.1%), exercise (223/307, 72.6%), and medication adherence (234/307,76.2%) Appropriateness of language used in SMS: 94.8% (291/307) Appropriateness of SMS frequency (4 times/week): 86.0% (264/307); timing : 89.9% (276/307, random timing was considered ideal); and 6-month duration: 77.2% (237/307)
	Feasibility	Not assessed
	Fidelity	96.0% (338/352) of participants received all scheduled messages (analytic data) and read ≥75% of SMS messages: 95.4% (293/307 self-report survey respondents)
	Implementation cost	US $0.10/SMS message (<US $10 per capita)
	Penetration	Not assessed
	Sustainability	Not assessed
**Dale, 2015 [42]**		
	Acceptability	Satisfaction with 24-week program duration was 79% (48/61) and with number of SMS messages was 84% (51/61) Recommend to other people: 90% (55/61)
	Adoption	98% (60/61) of participants initiated the SMS intervention ≥1 website login: 75% (46/61); median 3, range 0-100
	Appropriateness	90% (55/61) and 43% (26/61) of participants felt that SMS messages and the website were good cardiac rehabilitation (CR) delivery methods, respectively Appropriate number of SMS messages: 84% (51/61) Intervention useful for learning about (47/61, 77%) and recovering from (51/61, 84%) a heart event and for changing behaviors, such as physical activity (39/61, 64%) and consumption of fruit and vegetables (37/61, 61%), saturated fat (34/61, 56%), and salt (26/61, 43%)
	Feasibility	Not assessed
	Fidelity	Read all SMS messages: 85% (52/61) Sent ≥1 SMS step count message: 95% (58/61); mean of 15 submissions (SD 8.7) over 24 weeks
	Implementation cost	Not assessed
	Penetration	Not assessed
	Sustainability	Not assessed
**Kraal, 2013 [60,66]**		
	Acceptability	Satisfaction was higher for telerehabilitation than for center-based rehabilitation (8.7/10 vs 8.1/10; *P*=.02)
	Adoption	Not assessed
	Appropriateness	Not assessed
	Feasibility	Not assessed
	Fidelity	Exercise adherence was similar in telerehabilitation and center-based rehabilitation (mean 22.0, SD 6.8, vs mean 20.6, SD 4.3, sessions)
	Implementation cost	Similar per-capita cost to deliver telerehabilitation and center-based rehabilitation (€314 vs €336) Per-capita costs did not differ between telerehabilitation and center-based rehabilitation for total health care use (mean €2419, SD 1968, vs mean €2855, SD 2797; *P*=.39) or total work absenteeism (mean €3846, SD 8400, vs mean €6569, SD 8170; *P*=.12) Probability of cost-effectiveness was higher for telerehabilitation than for center-based rehabilitation under several assumptions
	Penetration	Not assessed
	Sustainability	Not assessed
**Lear, 2015 [61,67]**		
	Acceptability	22 purposively sampled interviews reported satisfaction, acceptability, and confidence in using virtual CR
	Adoption	High self-reported engagement and utilization in virtual CR (interview data) Mean website log-ins was 27 per participant (range 0-140) Mean engagement in chat sessions with health care providers was 3.6
	Appropriateness	Virtual CR perceived to be accessible and effective
	Feasibility	Virtual CR perceived to be convenient
	Fidelity	Not assessed
	Implementation cost	Not assessed
	Penetration	Not assessed
	Sustainability	Not assessed
**Maddison, 2015 [43,68]**		
	Acceptability	SMS and website intervention components were liked by 57% (43/75) and 73% (55/75) of participants, respectively Acceptability of 24-week intervention duration: 71% (53/75) Acceptability of message delivery timing: 57% (43/75); exercise ideas SMS content: 77% (58/75); exercise benefits education content: 79% (59/75); and website content: 47% (35/75); 49% (37/75) did not use the website
	Adoption	Not assessed
	Appropriateness	Some (number not reported) participants who were already exercising felt the intervention was unnecessary or the exercise prescription was not relevant
	Feasibility	Difficulties using website: 17% (13/75) Major barriers were lack of high-speed broadband or knowledge about using websites
	Fidelity	93% (70/75) read most SMS messages 64% (48/75) used the website (visits per participant: mean 11, SD 16, range 0-82)
	Implementation cost	NZ $239 per capita (intervention set-up + delivery only; health care utilization and indirect societal costs excluded) Incremental cost-effectiveness ratio: NZ $28,768 per quality-adjusted life year (QALY) Probability of cost-effectiveness: 72% (willingness to pay: NZ $20,000 per QALY) and 90% (willingness to pay: NZ $50,000 per QALY)
	Penetration	Not assessed
	Sustainability	Not assessed
**Maddison, 2019 [45,69]**		
	Acceptability	87% (58/67) would choose telerehabilitation instead of center-based rehabilitation if implemented in clinical practice Satisfaction with individualized exercise prescription: 90% (60/67); real-time exercise monitoring: 94% (63/67); encouragement and social support: 87% (58/67); behavior change messages: 85% (57/67); self-monitoring: 96% (64/67); and goal-setting features: 69% (46/67)
	Adoption	94% (77/82) of participants initiated telerehabilitation
	Appropriateness	97% (65/67) of patients reported that telerehabilitation is a good approach for delivering exercise rehabilitation
	Feasibility	Wearable sensor is easy to use: 99% (66/67); and is comfortable: 97% (65/67) Smartphone app is easy to use: 79% (53/67); easy to understand: 87% (58/67); and reliable: 66% (44/67) Rare technical difficulties, commonly solved with familiarization
	Fidelity	Adherence to prescribed exercise was comparable in telerehabilitation (mean 58.34%, SD 36.58, range 0-100) and center-based rehabilitation (mean 63.80%, SD 30.59, range 0-100; *P*=.31)
	Implementation cost	Lower per-capita program delivery cost for telerehabilitation than for center-based rehabilitation (NZ $1130 vs NZ $3466) No difference in total (ie, program delivery + health care and medication utilization) per-capita cost (NZ $4920 vs NZ $9535)
	Penetration	Not assessed
	Sustainability	Not assessed
**Park, 2014 [62]**		
	Acceptability	Strong or moderate agreement about intervention satisfaction: 82% (23/28) for SMS reminders + education; and 88% (22/25) for SMS education alone
	Adoption	Not assessed
	Appropriateness	Strong or moderate agreement that the interventions were useful for assisting medication adherence: 71% (20/28) for SMS reminders + education; and 48% (12/25) for SMS education alone
	Feasibility	Strong or moderate agreement that interventions were easy to use: 88.6% Technical difficulties receiving SMS: 7.6%
	Fidelity	Not assessed
	Implementation cost	Not assessed
	Penetration	Not assessed
	Sustainability	Not assessed
**Piotrowicz, 2014 [63,70]**		
	Acceptability	Not assessed
	Adoption	Not assessed
	Appropriateness	Felt safer during exercise with hybrid telerehabilitation than unsupervised: 80.9% Hybrid telerehabilitation was useful for increasing exercise: 95%; daily physical activity: 80%; and mental health: 71%
	Feasibility	Telemonitoring device was very easy or easy to use: 98.3% No problems self-fitting electrocardiogram (ECG) electrodes: 99.4% No problems transmitting ECG from home: 84% Missed ≥1 exercise session due to technical difficulties: 39.3% Problems communicating with telemonitoring center: 62.8%
	Fidelity	Not assessed
	Implementation cost	Not assessed
	Penetration	Not assessed
	Sustainability	Not assessed
**Salvi, 2018 [64,71]**		
	Acceptability	Guided exercise telerehabilitation ratings (mean [95% CI] rating score, max 5) for ease of use: 3.53 (2.94-4.12); interest: 4.42 (4.11-4.74); stimulation: 3.95 (3.49-4.41); and enjoyment: 3.84 (3.46-4.22) nb: data represent only 35% (19/55) of participants randomized to telerehabilitation
	Adoption	73% (40/55) of participants initiated guided exercise telerehabilitation Nonadoption was attributed to unavailability of the clinical team
	Appropriateness	Guided exercise telerehabilitation ratings (mean [95% CI] rating score, max 5) for usefulness to increase motivation: 4.59 (4.35-4.83); to increase safety: 4.47 (4.13-4.81); and to increase compliance: 4.47 (3.93-5.01) Overall, guided exercise telerehabilitation was considered appropriate for its purpose
	Feasibility	Exercise sessions affected by technical errors: 18% (ie, poor biosensor signal or connectivity and poor transmission of data to server) Suboptimal internet connectivity prevented 15 participants from recording or completing any exercise sessions 6 dropouts were attributed to technical challenges
	Fidelity	Participants initiated (mean [95% CI]) 61% (76%-46%) of the prescribed number of exercise sessions (79% [91%-67%] among 17 participants who completed the study) and completed 32% (44%-20%) of the prescribed duration of exercise (45% [59%-31%] among 17 participants who completed the study)
	Implementation cost	Not assessed
	Penetration	Not assessed
	Sustainability	Not assessed
**Varnfield, 2014 [44]**		
	Acceptability	Not assessed
	Adoption	Program uptake (ie, completion of ≥1 exercise session) was higher in telerehabilitation than center-based rehabilitation: 80% (48/60) vs 62% (37/60); relative risk (RR)=1.30, 95% CI 1.03-1.64; *P*<.05
	Appropriateness	Smartphone-measured step counts increased motivation to reach exercise goals: 84% (38/45)
	Feasibility	Not assessed
	Fidelity	Categorical adherence (ie, completing 4/6 weeks of exercise training) was higher in telerehabilitation than center-based rehabilitation: 95% (45/48) vs 68% (25/37); RR=1.40, 95% CI 1.13-1.70; *P*<.05
	Implementation cost	Not assessed
	Penetration	Not assessed
	Sustainability	Not assessed

#### Acceptability

Out of 10 included studies, 8 (80%) [41-43,45,60-62,64] reported the acceptability of telerehabilitation interventions from the participant perspective only; none reported acceptability from the individual provider or organization perspectives. Specific outcome measures within the acceptability implementation construct included perceived acceptability, satisfaction, likes and dislikes, interest, stimulation, and enjoyment. Studies reported high rates of acceptance for cardiac telerehabilitation, ranging from 71% [43] to 99% of participants [45]. Interventions that facilitated interaction between participants and providers [42,45,60,61,64] and delivered individually tailored content [43,60], in particular, appeared to have high acceptability. However, 4 studies out of 10 (40%) reported lack of interest among some participants [43,62,64]. In particular, messaging interventions (eg, SMS and push notifications) were not satisfactory for participants who would prefer face-to-face interaction with rehabilitation professionals [43]. Usability challenges such as insufficient internet connectivity, which fall within the *feasibility* implementation construct (see Feasibility section below), can also impact negatively on acceptability [42,64].

#### Adoption

Out of 10 included studies, 6 (60%) reported adoption of the intervention from the participant perspective only; none reported adoption from the individual provider or organization perspectives [41,42,44,45,61,64]. Specific outcome measures within the adoption implementation construct included initial uptake and engagement with telerehabilitation interventions. While levels of adoption varied between studies, they were generally high across SMS, website, and smartphone-based interventions. Out of 10 studies, 1 (10%) attributed some lack of adoption to the availability of staff delivering the intervention [64].

#### Appropriateness

Out of 10 included studies, 9 (90%) reported on the appropriateness of cardiac telerehabilitation from the participant perspective; none reported on appropriateness from the individual provider or organization perspectives [41-45,61-64]. Studies included a very broad range of outcome measures within the appropriateness implementation construct, including usefulness; suitability as an alternative CR delivery model; perceptions of safety, reassurance, accessibility, and effectiveness; as well as appropriateness of the intervention content, language, frequency, and duration.

These outcomes were positively appraised by the majority of participants, and cardiac telerehabilitation was perceived as convenient, flexible, safe, instant, private, and user-friendly. Many participants considered messaging (eg, SMS and push notifications) and smartphone apps to be appropriate mechanisms for delivering CR support [41,42,45,61,63]. Comprehensibility and interactivity also appeared to support participants’ perceptions of intervention appropriateness. [41-43,45,60,61,63,64]. Participants reported that telerehabilitation interventions were useful for increasing motivation and confidence to exercise, modifying health behaviors such as medication adherence and healthy eating, self-monitoring their health condition, and facilitating remote access to individualized exercise support [41,42,45,61,62,64]. Small proportions of participants perceived SMS to be either inadequate or unnecessary [41-43,62]. Overall, cardiac telerehabilitation interventions were considered appropriate by most participants [41-45,61-64]; however, significant heterogeneity of intervention designs and outcome measures in the literature we reviewed makes it difficult to identify factors that optimize intervention appropriateness.

#### Feasibility

Out of 10 included studies, 6 (60%) reported aspects of feasibility from a participant perspective but not from the individual provider or organization perspectives. Specific outcome measures within the feasibility implementation construct included usability, suitability of interventions for everyday use among participants, system reliability, or technical difficulties experienced by participants [43,45,61-64]. No included studies assessed the feasibility of telerehabilitation delivery from individual provider or organization perspectives.

Large majorities of telerehabilitation participants self-reported that technologies such as SMS, wearable sensors, smartphone apps, and websites were easy to use, convenient, comfortable, and easy to understand [43,45,61-63]. However, some technical challenges were noted. Approximately 20%-30% of participants reported reliability issues during real-time, remotely monitored, exercise rehabilitation, although the authors did not report whether issues were related to the required smartphone app, wearable sensor, or broadband internet connection [45]. Out of 10 studies, 4 (40%) reported a negative impact of unreliable broadband connectivity on user experiences during interventions that included web-based components and/or transmission of data to CR providers, which, at worst, can prevent participants from initiating their telerehabilitation intervention at all [43,62-64].

#### Fidelity

Out of 10 included studies, 7 (70%) reported on the fidelity of intervention receipt and/or completion among participants [41-45,60,64]. Specific outcome measures within the fidelity implementation construct included participant responsiveness or adherence to the intervention, such as receiving all the scheduled SMS messages, program completion, and adherence to prescribed exercise or medication. Large majorities (≥75%) of participants in SMS interventions self-reported reading all or most messages [41-43]. However, self-reported use of an intervention website was lower [43]. Out of 10 studies, 3 (30%) demonstrated that adherence to prescribed exercise telerehabilitation sessions was comparable to [45,60], or better than [44], center-based comparators.

Telerehabilitation interventions appeared to be delivered as per study protocols, with the exception of deviations caused by technical challenges (see Feasibility section above); however, only 1 study out of 10 (10%) formally assessed the fidelity of intervention delivery; analytic data indicated all SMS messages were successfully delivered to 96% of participants [41].

#### Implementation Cost

Only 4 of 10 (40%) included studies reported analyses of telerehabilitation intervention cost [41,43,45,60]. As interventions appear to have been provided at no cost to participants, these data likely represent cost from an organizational perspective. Specific outcome measures within this implementation construct included intervention delivery cost and cost-effectiveness. Intervention delivery costs varied markedly from US $10 per capita for an SMS intervention [41] to NZ $1130 per capita for a smartphone-based intervention that delivered real-time remote exercise supervision and coaching [45]. Out of 10 studies, 1 (10%) reported that the telerehabilitation intervention delivery cost was comparable to center-based CR [60], while another (1/10, 10%) reported almost 70% lower delivery costs for telerehabilitation compared with center-based programs [45]. Out of 10 studies, 2 (20%) that conducted cost-effectiveness analyses reported a 72%-90% probability of cost-effectiveness for an SMS intervention, assuming willingness-to-pay thresholds of NZ $20,000-$50,000 per quality-adjusted life year [43], and moderate to high probabilities that telerehabilitation would be more cost-effective than center-based rehabilitation, particularly at low willingness-to-pay thresholds [60]. None of the studies included in this review evaluated how telerehabilitation could be funded as an adjunct to existing CR services (ie, in additional to center-based program delivery costs).

#### Penetration and Sustainability

No included studies assessed any outcome measures within the penetration or sustainability implementation constructs.

## Discussion

### Principal Findings

The primary finding of our review is that, despite encouraging evidence for effectiveness [33-37], there is a lack of evidence evaluating the translation of cardiac telerehabilitation interventions from research into routine clinical practice. It is unclear whether this suggests that evidence-based interventions have yet to be implemented in clinical practice, have been implemented without evaluation, or have been implemented and evaluated but not yet published in the scientific literature.

The next best available data comes from a small number of experimental studies that have assessed key constructs related to the effective implementation of evidence-based interventions. Almost all included studies reported factors related to intervention appropriateness, acceptability, and fidelity; however, adoption, fidelity, and cost have received less attention, and intervention penetration and sustainability had yet to be evaluated.

Moreover, while consumers (ie, CR participants), individual providers (ie, CR practitioners), and organizations (ie, health care services) all play critical roles in achieving successful implementation outcomes [54,72], the cardiac telerehabilitation literature we reviewed has focused only on the consumer perspective. This may reflect the lack of research conducted in routine clinical practice, as individual and organizational providers may have little involvement in the delivery or management of telerehabilitation interventions during experimental trials.

Cardiac telerehabilitation was generally well accepted among the majority of participants, even across a broad range of different interventions. While the small number of studies in our review makes it difficult to determine which interventions may be most acceptable to participants, intervention features that enable participants to communicate with practitioners and receive tailored or individualized support appear to promote high rates of acceptance. Unfortunately, acceptability has not yet been evaluated from a delivery perspective, so the perceptions of rehabilitation providers (ie, individual practitioners and organizations) remain unknown. Cardiac telerehabilitation was considered an appropriate delivery model by many participants, particularly those who value convenient, flexible, and accessible intervention support. Moreover, many participants reported that cardiac telerehabilitation was useful for improving their self-management of lifestyle behaviors and CVD risk factors. At the participant level, acceptability and appropriateness also appear to be moderated by intervention feasibility. Interventions that were simple to access, easy to use, reliable, and delivered through ubiquitous mobile, smartphone, and/or web technologies appeared to have higher acceptability and appropriateness.

As our review findings are drawn from experimental studies, it is unclear if they would generalize to the delivery of cardiac telerehabilitation in routine clinical practice. In particular, the predominance of randomized treatment allocation in included studies differs markedly from a recommendation that CR participants should be offered a choice of alternative CR delivery models that best fit their needs and preferences [73]. Studies that include preference-based treatment allocation are needed to mimic this key element of routine clinical practice or, better yet, evaluation studies should be conducted in parallel with the translation of cardiac telerehabilitation into routine clinical practice. The single non-RCT included in the review reported high acceptance and usability among participants who preferred telerehabilitation [63].

Reporting of participant-level feasibility was mixed, which may reflect the difficulty of documenting both the incidence and impact of usability and technical challenges. However, collectively the evidence suggests telerehabilitation is feasible for most participants. Feasibility among individual and organizational providers is also critical for successful implementation [74,75] but, similar to other implementation constructs, was not evaluated in the included studies.

Fidelity is one of the important implementation outcomes, as it contributes to intervention quality [54]. While 7 of 10 (70%) included studies reported high intervention uptake, some outcome measures of adherence may be confounded by self-reporting bias. Reassuringly, 3 (30%) studies indicate that adherence to cardiac telerehabilitation can be at least as high as center-based programs [44,45,60]. A key gap in the literature we reviewed is the lack of information about the fidelity of intervention delivery from the perspectives of individual and organizational providers, which is critical to understand when implementing new interventions to maintain intervention quality [76]. This may reflect known challenges in measuring implementation constructs and a lack of available validated tools [54]; however, we note that the delivery fidelity of interventions that require little, if any, provider input (eg, SMS) may be sufficiently assessed via software analytics.

There was little specific evidence that intervention delivery deviated from study protocols in the included studies, but it is unclear whether this indicates high fidelity intervention delivery or a lack of documentation to support such a conclusion. While future translational research could comprehensively evaluate the fidelity of intervention delivery, comparisons with preceding experimental research may be confounded by assumptions about equivalence between experimental and translational research contexts.

Intervention cost is a key contributor to low uptake of interventions by health care providers [77], and comparison of costs and cost-effectiveness is crucial for making evidence-based decisions about implementing and scaling new interventions [76]. Relatively few included studies reported economic analyses, and telerehabilitation costs varied markedly across different intervention designs. Our review indicates that telerehabilitation can reduce CR delivery costs and be cost-effective, but it is unclear if cost-effectiveness varies between different types of interventions. For example, interventions that require significant practitioner input, such as real-time remote exercise monitoring and coaching [45], may be substantially cheaper to deliver than center-based programs but more expensive than semiautomated interventions, such as SMS [43]. Whether or not an intervention represents good value depends on health effects and costs, as well as intervention and health care objectives. Therefore, examining the cost and health effects of telerehabilitation interventions in routine clinical practice is essential to provide more valuable information for implementation and scale-up.

The remaining implementation constructs of interest in this review—penetration and sustainability—were not assessed in any included studies. This was not surprising, as penetration and sustainability are more relevant to the mid- and later stages of implementation [54]. Short-duration experimental trials limit penetration to those who are willing to volunteer for research and preclude assessment of longer-term intervention sustainability. As a result, we lack valuable information on factors that could influence the sustainability of the interventions and how this might impact potential scale-up.

### Opportunities for Future Research

Evidence suggests the spread, scale-up, and sustainability of health care innovations are influenced by a broad range of factors related to the people who receive (ie, participants) and deliver (ie, health care practitioners and providers) the innovations as well as by numerous organizational and societal factors [55,56,74]. The experimental studies in our review focused on a relatively narrow range of outcomes, omitted the individual and organizational provider stakeholder levels, and could not replicate the complexity of implementation in routine clinical practice. Robust experimental evaluation of effectiveness and safety is critical before real-world implementation [78]. However, it is now critical to evaluate the implementation of proven interventions in routine clinical practice, preferably as a complementary adjunct to existing center-based programs, to incorporate the critical element of consumer choice that we and others have advocated [45,73]. Such studies should target all key implementation constructs across all relevant stakeholder levels, be embedded fully in routine clinical practice, and be evaluated for a sufficient duration to enable comprehensive assessment of all factors that contribute to successful, scalable, and sustainable implementation [74].

While it was beyond the scope of this review, an understanding of the relative importance of different factors on the implementation of telerehabilitation interventions in clinical practice is also needed. Although effective implementation is understood to be an interactive combination of factors [74], our review highlights the variability in the assessment and reporting of implementation constructs. It is unknown if this variability was due to, for example, researchers’ perceived importance of specific factors when selecting the study outcomes, evidence for the differential impact of implementation factors on outcomes, or the feasibility of evaluating multiple factors within a trial design. Improving the translation of interventions into routine clinical practice requires a greater understanding of the roles of implementation factors and consistent measurement of their impact. Research that explores the relative importance of such factors would greatly advance our ability to effectively scale interventions.

### Strengths and Limitations

A major strength of this review was the use of a robust systematic review methodology to understand a novel research area. Secondly, while broad variability of intervention designs across a small number of studies makes it difficult to determine how to optimize telerehabilitation for translation into clinical practice, it provides a broad overview of potential issues that could be associated with implementation and scale-up.

The findings of our review are primarily limited by a lack of studies that have evaluated cardiac telerehabilitation interventions when implemented in routine clinical practice. The assessment of implementation-related outcomes during controlled experimental studies provides some insight, but marked differences within the context of real-world rehabilitation service delivery limit their generalizability. Additionally, while promising early evidence for effectiveness, safety, acceptability, and cost of cardiac telerehabilitation interventions [33-37] suggest they could play an important role in increasing overall participation in CR, it remains unclear whether positive trial outcomes will be retained following translation into clinical practice [76]. Our review includes a small number of studies with relatively small sample sizes and homogenous cohorts. This may limit generalizability to population subgroups who are typically underserved by CR, including older adults, women, people living in regional or rural areas, and people with diverse non-English-speaking cultural backgrounds [41-45,60,64]. Finally, there was a lack of studies from developing countries where telerehabilitation could have an even greater impact due to a low provision of traditional center-based CR [79-81].

### Conclusions

Cardiac telerehabilitation interventions appear to be acceptable and appropriate for many participants in experimental trials and may be a cost-effective way to increase the reach and utilization of CR. However, explicit implementation studies are urgently needed to inform best-practice translation into routine clinical practice. When possible, such studies should implement telerehabilitation in parallel with existing center-based programs so consumers can autonomously match program delivery models to their individual needs and preferences.

## Appendix

Multimedia Appendix 1 Search strategies for included studies.

Multimedia Appendix 2 Characteristics of included studies.

## Abbreviations

CHD: coronary heart disease
CR: cardiac rehabilitation
CVD: cardiovascular disease
ICT: information and communication technology
MI: myocardial infarction
PRISMA: Preferred Reporting Items for Systematic Reviews and Meta-Analyses
PROSPERO: International Prospective Register of Systematic Reviews
RCT: randomized controlled trial
