# Supplementary material for: Implementation of Telerehabilitation Interventions for the Self-Management of Cardiovascular Disease: Systematic Review
Source: JMIR Mhealth Uhealth. 2020 Nov 27;8(11):e17957. doi: 10.2196/17957 (PMC7732711; doi:10.2196/17957)
Supplement: Multimedia Appendix 1 [file mhealth_v8i11e17957_app1.docx]

# Multimedia Appendix 1: Search Strategies

## OVID Medline

| **#** | **Search terms** | **Results** |
| --- | --- | --- |
| 1 | Exp Telemedicine/ OR exp Telemetry/ OR exp Telephone/ OR exp Wireless Technology/ OR exp Computers, Handheld/ OR exp Internet/ | 124476 |
| 2 | (telemedicine OR telemetry OR telehealth OR telecare OR telerehab* OR telemonitor* OR e?health OR m?health OR mobile health).tw. | 23625 |
| 3 | ((tele* OR remote OR mobile) ADJ3 (medicine OR health OR monitor* or rehab*)).tw. | 11780 |
| 4 | (smart?phone OR cell?phone OR telephone OR Videoconferencing OR Text Messaging OR Instant Messaging OR ((cell* OR mobile OR smart OR wireless) AND (phone OR device OR technology))).tw. | 148951 |
| 5 | (internet OR website OR web?based OR online).tw. | 133530 |
| 6 | ((mobile OR phone OR android OR apple OR ios) AND (app* OR software)).tw. | 54127 |
| 7 | 1 OR 2 OR 3 OR 4 OR 5 OR 6 | 400891 |
| 8 | Exp Myocardial Ischemia/ OR exp Coronary Disease/ OR exp Angina Pectoris/ OR exp Acute Coronary Syndrome/ OR exp Myocardial Infarction/ | 407268 |
| 9 | ((Myocardial OR heart) ADJ3 (infarct* OR attack OR isch?em*)).tw. | 241662 |
| 10 | ((coronary OR isch?em* OR acute) ADJ3 (disease OR bypass OR syndrome OR angioplast*) OR (percutaneous coronary intervention OR angina OR stent).tw. | 661369 |
| 11 | 8 OR 9 OR 10 | 839874 |
| 12 | Exp Evaluation Studies/ OR exp Program Evaluation/ | 300592 |
| 13 | (Evaluat* Stud* OR Program* Evaluat*).tw. | 12236 |
| 14 | ((scale AND (up OR out)) OR scaling OR scalab* OR (roll* AND out) OR rollout OR "real?world").tw. | 196385 |
| 15 | (translat* OR adopt* OR uptake OR utili?ation OR sustain* OR program* OR initiative* OR feasib*).tw. | 2220418 |
| 16 | ((program* OR intervention) ADJ5 (delivery OR uptake OR adopt* OR adapt* OR modif* OR translat* OR disseminat* OR implement* OR diffus*)).tw. | 68730 |
| 17 | ((translat* OR disseminat* OR implement* OR diffus* OR research) ADJ3 (framework* OR model* OR plan* OR approach* OR strateg* OR protocol* OR guideline* OR manual* OR concept* OR practice)).tw. | 164219 |
| 18 | 12 OR 13 OR 14 OR 15 OR 16 OR 17 | 2715519 |
| 19 | 7 and 11 and 18 | 1969 |
| 20 | limit 19 to (english language and  yr="1990 -Current") | 1799 |

## Ovid Embase

| **#** | **Search terms** | **Results** |
| --- | --- | --- |
| 1 | exp telemedicine/ or exp telemetry/ or exp telephone/ or wireless communication/ or exp personal digital assistant/ or exp internet/ | 190416 |
| 2 | (telemedicine or telemetry or telehealth or telecare or telerehab* or telemonitor* or e?health or m?health or mobile health).tw. | 31969 |
| 3 | ((tele* or remote or mobile) adj3 (medicine or health or monitor* or rehab*)).tw. | 16206 |
| 4 | (smart?phone or cell?phone or telephone or videoconferencing or text messaging or instant Messaging or ((Cell* or mobile or smart or wireless) and (phone or device or technology))).tw. | 207653 |
| 5 | (internet or website or web?based or online).mp. | 188282 |
| 6 | ((mobile or phone or android or apple or ios) and (app* or software)).tw. | 79308 |
| 7 | 1 OR 2 OR 3 OR 4 OR 5 OR 6 | 559699 |
| 8 | Exp heart muscle ischemia/ OR coronary artery disease/ OR exp angina pectoris/ OR exp coronary artery disease/ OR exp heart infarction/ | 686956 |
| 9 | ((myocardial or heart) adj3 (infarct* or attack or isch?em*)).tw. | 355375 |
| 10 | (((coronary or isch?em* or acute) adj3 (disease or bypass or syndrome or angioplast*)) or (percutaneous coronary intervention or angina or stent)).tw. | 551050 |
| 11 | 8 or 9 or 10 | 989730 |
| 12 | exp evaluation study/ or exp Program evaluation/ | 53475 |
| 13 | (Evaluat* Stud* or Program* Evaluat*).tw. | 16295 |
| 14 | ((scale and (up or out)) or scaling or scalab* or (roll* and out) or rollout or "real?world").tw. | 260566 |
| 15 | (translat* or adopt* or uptake or utili?ation or sustain* or program* or initiative* or feasib*).tw. | 2940529 |
| 16 | ((program* or intervention) adj5 (delivery or uptake or adopt* or adapt* or modif* or translat* or  disseminat* or implement* or diffus*)).tw. | 90992 |
| 17 | ((translat* or disseminat* or implement* or diffus* or research) adj3 (framework* or model* or plan* or approach* or strateg* or protocol* or guideline* or manual* or concept* or practice)).tw. | 212986 |
| 18 | 12 or 13 or 14 or 15 or 16 or 17 | 3327200 |
| 19 | 7 and 11 and 18 | 3077 |
| 20 | limit 19 to (human and english language and yr="1990 -Current") | 2480 |
| 21 | Limit 20 to exclude medline journals | 135 |

## OVID PsycINFO

| **#** | **Search terms** | **Results** |
| --- | --- | --- |
| 1 | exp telemedicine/ or exp telemetry/ or exp telephone systems/ or mobile devices/ or exp computer assisted therapy/ or exp internet/ | 39939 |
| 2 | (telemedicine or telemetry or telephone systems or mobile devices or computer assisted therapy or internet or telehealth or telecare or telerehab* or telemonitor* or e?health or m?health or mobile health).tw. | 43194 |
| 3 | ((tele* or remote or mobile) adj3 (medicine or health or monitor* or rehab*)).tw. | 2761 |
| 4 | (smart?phone or cell?phone or telephone or videoconferencing or test messaging or instant messaging or ((cell* or mobile or smart or wireless) and (phone or device or technology))).tw. | 35021 |
| 5 | (internet or website or web?based or online).tw. | 97459 |
| 6 | ((mobile or phone or android or apple or ios) and (app* or software)).tw. | 11348 |
| 7 | 1 or 2 or 3 or 4 or 5 or 6 | 144180 |
| 8 | exp myocardial infarctions/ OR exp ischemia/ OR exp angina pectoris/ | 10823 |
| 9 | ((Myocardial or heart) adj3 (infarct* or attack or isch?em*)).tw. | 6131 |
| 10 | (((coronary or isch?em* or acute) adj3 (disease or bypass or syndrome or angioplast*)) or (percutaneous coronary intervention or angina or stent)).tw. | 11331 |
| 11 | 8 or 9 or 10 | 22540 |
| 12 | exp evaluation/ | 102746 |
| 13 | (evaluat* stud* or program* evaluat*).tw. | 13501 |
| 14 | ((scale and (up or out)) or scaling or scalab* or (roll* and out) or rollout or "real?world").tw. | 53984 |
| 15 | (translat* or adopt* or uptake or utili?ation or sustain* or program* or initiative* or feasib*).tw. | 640163 |
| 16 | ((program* or intervention) adj5 (delivery or uptake or adopt* or adapt* or modif* or translat* or disseminat* or implement* or diffus*)).tw. | 40417 |
| 17 | ((translat* or disseminat* or implement* or diffus* or research) adj3 (framework* or model* or plan* or approach* or strateg* or protocol* or guideline* or manual* or concept* or practice)).tw. | 117061 |
| 18 | 12 or 13 or 14 or 15 or 16 or 17 | 833998 |
| 19 | 7 and 11 and 18 | 195 |
| 20 | limit 19 to (peer reviewed journal and english language and yr="1990 -Current") | 145 |

## Global Health

| **SN** | **Search terms** | **Results** |
| --- | --- | --- |
| 1 | ["telemedicine" OR "telemetry" OR "telephones" OR "computers" OR "internet"](https://www-cabdirect-org.ezproxy.lib.monash.edu.au/cabdirect/search/?q=%22telemedicine%22%20OR%20%22telemetry%22%20OR%20%22telephones%22%20OR%20%22computers%22%20OR%20%22internet%22&sort=Relevance) | 136,384 |
| 2 | [telemedicine OR telemetry OR telehealth OR telecare OR telerehab* OR telemonitor* OR ehealth OR mhealth OR mobile health](https://www-cabdirect-org.ezproxy.lib.monash.edu.au/cabdirect/search/?q=telemedicine%20OR%20telemetry%20OR%20telehealth%20%20OR%20telecare%20%20OR%20telerehab*%20OR%20telemonitor*%20%20OR%20ehealth%20%20OR%20%20mhealth%20OR%20%20mobile%20health&sort=Relevance) | 9,630 |
| 3 | ["smartphones" OR "cellphones" OR "mobile phones" OR "mobile telephones"](https://www-cabdirect-org.ezproxy.lib.monash.edu.au/cabdirect/search/?q=%22smartphones%22%20OR%20%22cellphones%22%20OR%20%22mobile%20phones%22%20OR%20%22mobile%20telephones%22&sort=Relevance) | 4,209 |
| 4 | [videoconferencing OR text messaging OR instant messaging](https://www-cabdirect-org.ezproxy.lib.monash.edu.au/cabdirect/search/?q=videoconferencing%20%20OR%20text%20messaging%20%20OR%20instant%20messaging&sort=Relevance) | 694 |
| 5 | ["mobile application*" OR "mobile app*" OR "smartphone app*" OR "smartphone application*" OR "mobile software*" OR "android app*" OR "android application" OR "ios app*" OR "ios application*"](https://www-cabdirect-org.ezproxy.lib.monash.edu.au/cabdirect/search/?q=%22mobile%20application*%22%20%20OR%20%22mobile%20app*%22%20%20OR%20%22smartphone%20app*%22%20%20OR%20%20%22smartphone%20application*%22%20%20OR%20%20%22mobile%20software*%22%20%20OR%20%20%22android%20app*%22%20%20OR%20%22android%20application%22%20%20OR%20%22ios%20app*%22%20%20OR%20%20%22ios%20application*%22&sort=Relevance) | 965 |
| 6 | 1 or 2 or 3 or 4 or 5 | 143,317 |
| 7 | ["myocardial infarction" OR "myocardial ischaemia" OR "coronary diseases"](https://www-cabdirect-org.ezproxy.lib.monash.edu.au/cabdirect/search/?q=%22myocardial%20infarction%22%20OR%20%22myocardial%20ischaemia%22%20OR%20%22coronary%20diseases%22&sort=Relevance) | 55,534 |
| 8 | ["angina pectoris" OR "acute coronary syndrome" OR "myocardial attack" OR "heart attack" OR "coronary bypass" OR "coronary syndrome" OR angioplast* or "percutaneous coronary intervention" OR "ischaemic disease*" OR "ischemic disease*"](https://www-cabdirect-org.ezproxy.lib.monash.edu.au/cabdirect/search/?q=%22angina%20pectoris%22%20%20OR%20%22acute%20coronary%20syndrome%22%20%20OR%20%22myocardial%20attack%22%20OR%20%22heart%20attack%22%20%20OR%20%22coronary%20bypass%22%20%20OR%20%22coronary%20syndrome%22%20%20OR%20angioplast*%20or%20%22percutaneous%20coronary%20intervention%22%20OR%20%22ischaemic%20disease*%22%20%20OR%20%22ischemic%20disease*%22&sort=Relevance) | 12,130 |
| 9 | 7 or 8 | 56,925 |
| 10 | ["evaluation" OR "program evaluation" OR "implementation" OR "implementation of research" OR "scaling" OR "sustainability" OR "translation" OR "adoption" OR "adoption of innovations" OR "uptake" OR "feasibility" OR "feasibility studies" OR "utilization" OR "diffusion" OR "program development" OR "program effectiveness"](https://www-cabdirect-org.ezproxy.lib.monash.edu.au/cabdirect/search/?q=%22evaluation%22%20OR%20%22program%20evaluation%22%20OR%20%22implementation%22%20OR%20%22implementation%20of%20research%22%20OR%20%22scaling%22%20OR%20%22sustainability%22%20OR%20%22translation%22%20OR%20%22adoption%22%20OR%20%22adoption%20of%20innovations%22%20OR%20%22uptake%22%20OR%20%22feasibility%22%20OR%20%22feasibility%20studies%22%20OR%20%22utilization%22%20OR%20%22diffusion%22%20OR%20%22program%20development%22%20OR%20%22program%20effectiveness%22&sort=Relevance) | 1,694,376 |
| 11 | ["scale up" OR "scale out" OR scal* OR "roll out" OR "rollout" OR "real world" OR "program uptake" OR "program delivery" OR "program adoption" OR "program adap*" OR "program implemen*" OR "intervention diffusion" OR translat*](https://www-cabdirect-org.ezproxy.lib.monash.edu.au/cabdirect/search/?q=%22scale%20up%22%20%20OR%20%22scale%20out%22%20%20OR%20scal*%20%20OR%20%22roll%20out%22%20%20OR%20%22rollout%22%20OR%20%22real%20world%22%20%20OR%20%22program%20uptake%22%20%20OR%20%22program%20delivery%22%20%20OR%20%22program%20adoption%22%20%20OR%20%22program%20adap*%22%20%20OR%20%22program%20implemen*%22%20%20OR%20%22intervention%20diffusion%22%20%20OR%20translat*&sort=Relevance) | 517,620 |
| 12 | 10 or 11 | 1,772,285 |
| 13 | 6 and 9 and 12 | 167 |
| 14 | Limit to [yr:[1990 TO 2018]](https://www-cabdirect-org.ezproxy.lib.monash.edu.au/cabdirect/search/?q=((%22scale%20up%22%20%20OR%20%22scale%20out%22%20%20OR%20scal*%20%20OR%20%22roll%20out%22%20%20OR%20%22rollout%22%20OR%20%22real%20world%22%20%20OR%20%22program%20uptake%22%20%20OR%20%22program%20delivery%22%20%20OR%20%22program%20adoption%22%20%20OR%20%22program%20adap*%22%20%20OR%20%22program%20implemen*%22%20%20OR%20%22intervention%20diffusion%22%20%20OR%20translat*)%20OR%20(%22evaluation%22%20OR%20%22program%20evaluation%22%20OR%20%22implementation%22%20OR%20%22implementation%20of%20research%22%20OR%20%22scaling%22%20OR%20%22sustainability%22%20OR%20%22translation%22%20OR%20%22adoption%22%20OR%20%22adoption%20of%20innovations%22%20OR%20%22uptake%22%20OR%20%22feasibility%22%20OR%20%22feasibility%20studies%22%20OR%20%22utilization%22%20OR%20%22diffusion%22%20OR%20%22program%20development%22%20OR%20%22program%20effectiveness%22))%20AND%20((%22angina%20pectoris%22%20%20OR%20%22acute%20coronary%20syndrome%22%20%20OR%20%22myocardial%20attack%22%20OR%20%22heart%20attack%22%20%20OR%20%22coronary%20bypass%22%20%20OR%20%22coronary%20syndrome%22%20%20OR%20angioplast*%20or%20%22percutaneous%20coronary%20intervention%22%20OR%20%22ischaemic%20disease*%22%20%20OR%20%22ischemic%20disease*%22)%20OR%20(%22myocardial%20infarction%22%20OR%20%22myocardial%20ischaemia%22%20OR%20%22coronary%20diseases%22))%20AND%20((%22mobile%20application*%22%20%20OR%20%22mobile%20app*%22%20%20OR%20%22smartphone%20app*%22%20%20OR%20%20%22smartphone%20application*%22%20%20OR%20%20%22mobile%20software*%22%20%20OR%20%20%22android%20app*%22%20%20OR%20%22android%20application%22%20%20OR%20%22ios%20app*%22%20%20OR%20%20%22ios%20application*%22)%20OR%20(videoconferencing%20%20OR%20text%20messaging%20%20OR%20instant%20messaging)%20OR%20(%22smartphones%22%20OR%20%22cellphones%22%20OR%20%22mobile%20phones%22%20OR%20%22mobile%20telephones%22)%20OR%20(telemedicine%20OR%20telemetry%20OR%20telehealth%20%20OR%20telecare%20%20OR%20telerehab*%20OR%20telemonitor*%20%20OR%20ehealth%20%20OR%20%20mhealth%20OR%20%20mobile%20health)%252) and English | 150 |
