# Supplementary material for: Implementation of Telerehabilitation Interventions for the Self-Management of Cardiovascular Disease: Systematic Review
Source: JMIR Mhealth Uhealth. 2020 Nov 27;8(11):e17957. doi: 10.2196/17957 (PMC7732711; doi:10.2196/17957)
Supplement: Multimedia Appendix 2 [file mhealth_v8i11e17957_app2.docx]

**Multimedia Appendix 2: Characteristics of included studies**

**Chow 2015, (41, 65)**

| Design | | Two arm parallel, single-blind RCT | | | |
| --- | --- | --- | --- | --- | --- |
| Protocol/trial registration | | Yes/Yes (ACTRN12611000161921) | | | |
| Participants  Eligibility | | n=710 (intervention=352, control=358). Mean age 57.6 ±9.2 years. 82% male.  **Inclusion**  ≥18 years, documented CHD (MI, coronary artery bypass graft surgery, percutaneous coronary intervention or 50% or greater stenosis in at least 1 major epicardial vessel on coronary angiography), able to provide informed consent.  **Exclusion**  No mobile phone, insufficient English language proficiency to read text messages. | | | |
| Treatments | | **Intervention**: TEXT ME  ICT: mobile phones  Content: Usual care (see Control, below) + semi-personalized non-interactive SMS targeting smoking cessation, physical activity, healthy diet, cardiovascular health (4 messages/week for 24 weeks).  **Control:** Usual care  Community follow-up + referral to inpatient CR. | | | |
| Outcomes | | **Primary:** Plasma level low-density lipoprotein cholesterol concentration at 6 months.  **Secondary:** systolic blood pressure, BMI, total cholesterol concentration, waist circumference, heart rate, total physical activity, smoking status, and the proportion of participants achieving guideline levels of modifiable risk factors.  **Implementation:** Acceptability, appropriateness, adoption, feasibility, fidelity, and implementation cost. Level of analysis: participant/consumer. | | | |
| Follow-up | | 6 months | | | |
| Country | | Australia | | | |
| Risk of bias | | Judgement | | Support for judgement | |
| Sequence generation | | Low risk | | "The random allocation sequence was in a uniform 1:1 allocation ratio with a block size of 8". | |
| Allocation concealment | | Low risk | | "…random allocation sequence...was concealed from study personnel.” | |
| Blinding of patient/personnel  Blinding – outcome assessment | | Low risk  Low risk | | “To maintain blinding of study personnel. ….”  "To maintain blinding of study personnel, patients were informed of their allocation in a text message sent after hospital discharge". | |
| Incomplete outcome data | | Unclear risk | | Low loss-to-follow-up but handling of missing data not reported. | |
| Intention-to-treat analysis | | Low risk | | "…all intervention evaluations were performed on the principle of intention to treat". | |
| Selective reporting | | Low risk | | Protocol is available and all pre-specified outcomes of interest are reported. | |
| Other sources of bias | | Low risk | | No other risks identified. | |

**Dale 2015 (42)**

| Design | Two-arm parallel RCT | |
| --- | --- | --- |
| Protocol/trial registration | Yes/Yes (ACTRN12613000901707) | |
| Participants  Eligibility | n=123 (intervention=61, control=62), Mean age 59.5±11.1 years. 81% male.  **Inclusion**  Adults diagnosed with CHD (MI, angina or revascularization), English speaking, internet literacy and access.  **Exclusion**  Participants with untreated ventricular tachycardia, severe heart failure, life-threatening coexisting disease with life expectancy less than 1 year and/or significant exercise limitations for reasons other than CHD. | |
| Treatments | **Intervention:** Text4Heart  ICT: mobile phone and supporting website, pedometer  Content: 24-week evidence and theory-based personalized text message program with access to supporting website delivered in additional to usual CR (see usual CR below). Interventions aimed to educate participants about their CVD risk factors and support them to make relevant lifestyle changes including smoking cessation, limiting alcohol consumption (<14 units of alcohol/week), increasing fruit and vegetable intake (5 servings), decreasing salt consumption and start/maintain regular physical activity (150 minutes of moderate-to-vigorous intensity per week).  Seven messages delivered per week (1/day) and participants had access to a supporting website (biweekly tips, graph displaying step counts and short video messages). From 13 to 24 weeks, frequency of message decreased to 5/week. Participants self-monitored their physical activity using pedometer. Provision of bidirectional messaging (interaction with participants and providers).  **Control:** Center-based usual CR  1-hour outpatient education per week for 6 weeks at hospital or community center (CVD risk factors, lifestyle change, and psychosocial support) + 6 month (16-session) supervised exercise program. | |
| Outcomes | **Primary:** Participants adherence to recommended health guidelines at 6 months.  **Secondary:** Overall cardiovascular disease risk, body composition, illness perceptions, self-efficacy, hospital anxiety/depression and medication adherence  **Implementation:** Acceptability, appropriateness, adoption, fidelity. Level of analysis: provider/consumer. | |
| Follow-up | 3 months and 6 months | |
| Country | New Zealand | |
| Risk of bias | Judgement | Support for judgement |
| Sequence generation | Low risk | “…randomization sequence was computer generated by a statistician independent to the project using a block size of 6”. |
| Allocation concealment | Low risk | "Allocation was concealed in sequentially numbered, opaque, sealed envelopes". |
| Blinding of participants/personnel  Blinding – outcome assessment | High risk  High risk | "…participants and outcome assessors were not blinded to their treatment allocation".  "…participants and outcome assessors were not blinded to their treatment allocation". |
| Incomplete outcome data | Low risk | "Missing data were not imputed if the proportion of missing in the primary outcome was less than 10%." |
| Intention-to-treat analysis | Low risk | “The primary analysis was conducted on an intention to-treat basis, while a sensitivity analysis compared primary outcomes between ‘as treated’ groups”. |
| Selective reporting | Low risk | Had the protocol published and all the outcomes (primary and secondary outcomes) were reported. |
| Other sources of bias | High risk | “…primary outcome measure was self-reported, so recall bias is possible, although validated questionnaires were used where feasible”. |

**Kraal 2013 (60, 66)**

| Design | Two arm parallel RCT | |
| --- | --- | --- |
| Protocol/trial registration | Yes/Yes (NCT01732419) | |
| Participants  Eligibility | n=90 (intervention=45, control=45), Mean age=59.2±8.5 years. 89% male.  **Inclusion**  Acute coronary syndrome (ACS; myocardial infarction or unstable angina) or a revascularization (percutaneous coronary intervention (PCI) or coronary artery bypass grafting (CABG)) with low to moderate risk for further events classified by a cardiologist; internet access + personal computer at home.  **Exclusion**  Ventricular arrhythmias or myocardial ischemia during the maximal exercise test at baseline; left ventricular ejection fraction below 45%; psychological, physical or cognitive impairments that prevented participation in exercise based CR. | |
| Treatments | **Intervention:** Fit@Home  ICT: heart rate monitor, (Garmin FR 70), web application (Garmin connect) and telephone  Content: 3 familiarization exercise sessions in outpatient clinic + 12-week telerehabilitation program (≥2 sessions/week) comprising asynchronous monitoring of exercise duration and intensity level (heart rate) by physical therapist + weekly telephone motivational interview. Fit@Home participants also received non-exercise usual care CR components [unspecified].  **Control:** Usual care CR (center-based exercise + other CR [unspecified] components.  12 weeks of group-based training in outpatient clinic (≥2 session/week) supervised by CR therapist | |
| Outcomes | **Primary:** Physical fitness and physical activity levels.  **Secondary:** Health related quality of life, psychosocial status  **Implementation:** Acceptability and fidelity (level of analysis: participant/consumer). Implementation cost (level of analysis: participant/consumer and provider). | |
| Follow-up | 12 weeks, 6 months and 1 year. | |
| Country | Netherlands | |
| Risk of bias | Judgement | Support for judgement |
| Sequence generation | Low risk | “Allocation is based on randomization with variable block size (two or four), performed with dedicated computer software by a researcher (NP) who is not present at the time of allocation”. |
| Allocation concealment  Blinding of participant/personnel | High risk  High risk | *"*By design, we are unable to blind participants for allocation*".*  Participants and personnel were not blinded |
| Blinding – outcome assessment | High risk | "In addition, the personnel supervising the cardiopulmonary exercise tests are not blinded for allocation...” |
| Incomplete outcome data | Low risk | "We imputed missing cost and effect data using the Multivariate Imputation by Chained Equations (MICE) algorithm with 20 iterations" |
| Intention-to-treat analysis | Low risk | “The primary analysis was conducted on an intention to-treat basis, while a sensitivity analysis compared primary outcomes between ‘as treated’ groups”. |
| Selective reporting | Low risk | All the outcomes (primary and secondary outcomes reported as per protocol. |
| Other sources of bias | High risk | “…patients included in the study were not representative of the general population, mainly young and motivated patients that preferred to participate were included”. |

**Lear 2015 (61, 67)**

| Design | | Two arm parallel RCT | | | |
| --- | --- | --- | --- | --- | --- |
| Protocol/trial registration | | No/Yes (NCT00683813) | | | |
| Participants  Eligibility | | n=78, (intervention=38, control=40). Mean age intervention=61.7, control= 58.4. 85% male.  **Inclusion:** Cardiac inpatients (admitted for either acute coronary syndrome or revascularisation procedure) from two hospitals in British Columbia, low or moderate risk, regular internet access (home, work or other environment), no physical limitations for regular PA and fluent in English.  **Exclusion:** Patients already using CR, having depression, uncontrolled diabetes and other significant co-morbidities, pregnant women and those excluded by attending physician as unsuitable for participation. | | | |
| Treatments | | **Intervention:** Virtual cardiac rehabilitation (vCRP)  ICT: website, off-the-shelf heart rate monitor (Polar s610i) and home BP monitor (Lifesource UA779)  Content: Asynchronous monitoring of blood pressure, and heart rate monitoring during exercise with transfer of data to vCRP webserver. On-line intake (medical, risk factor and lifestyle assessment), interactive one-on-one chat sessions.  **Control:** Usual care  Care from primary care physician + exercise and health eating guidelines + list of internet-based resources | | | |
| Outcomes | | **Primary:** Exercise capacity (MET_peak_) during symptom-limited treadmill test (Bruce protocol)  **Secondary:** concentrations of total and high-density lipoprotein cholesterol, triglycerides, and blood glucose  **Implementation:** Appropriateness, acceptability, adoption and feasibility. Level of analysis: participant/consumer. | | | |
| Follow-up | | 4 and 12 months | | | |
| Country | | Canada | | | |
| Risk of bias | | Judgement | | Support for judgement | |
| Sequence generation | | High risk | | Sequence generation method not mentioned | |
| Allocation concealment  Blinding of participant/personnel | | Low risk  Low risk | | "The randomization research coordinator informed the participants of their group assignment…"  “…there was no contact between the study personnel and the usual care participants for the duration of the study…” | |
| Blinding – outcome assessment | | Low risk | | "…with blinded outcome assessment…" | |
| Incomplete outcome data | | Low risk | | No missing data | |
| Intention-to-treat analysis | | Low risk | | "…using intent-to-treat analysis." | |
| Selective reporting | | Low risk | | Not identified. | |
| Other sources of bias | | Low risk | | No other risks identified | |

**Maddison 2015 (43, 68)**

| Design | Two arm parallel RCT | |
| --- | --- | --- |
| Protocol/trial registration | Yes/Yes (ACTRN12611000117910) | |
| Participants  Eligibility | n=171 (intervention=85, control=86). Mean age = 60 ± 9 y. 81% male.  **Inclusion**  Angina pectoris, MI or revascularisation (PCI, CABG/stent) within 3-24 months; clinically stable outpatients; internet access; able to exercise; read/write English.  **Exclusion**  Heart disease-related hospital admission within 6 weeks; terminal cancer; significant non-coronary exercise limitations. | |
| Treatments | **Intervention:** HEART  ICT: SMS and website  Content: usual care + 24 week SMS exercise intervention aiming to encourage ≥ 30 min moderate to vigorous exercise on ≥ 5 days per week. SMS included regular personalized exercise prescription and behaviour change messages (motivation, self-efficacy, social support). Messages frequency = 6 per week (1-3 months), 5 per week (3-4.5 months), 4 per week (4.5-6 months). Website featuring self-monitoring tools, video messages, lifestyle and CVD risk education, and links to relevant organizations' websites.  **Control:** Usual care CR  Access to community CR education sessions, encouragement to be physical active and join a cardiac club. | |
| Outcomes | **Primary:** Exercise capacity (V̇O_2_peak).  **Secondary:** Blood pressure, body composition, physical activity, health-related quality of life.  **Implementation:** Acceptability, appropriateness, feasibility, and implementation cost. Level of analysis: participant/consumer. | |
| Follow-up | 6 months | |
| Country | New Zealand | |
| Risk of bias | Judgement | Support for judgement |
| Sequence generation | Low risk | "…eligible participants were randomly allocated at a 1:1 ratio…by means of a central computerized service…using the minimization method…" |
| Allocation concealment | Low risk | "Allocation concealment was maintained up to the point of randomization." |
| Blinding of participant/personnel  Blinding – outcome assessment | High risk  Low risk | Trial participants were not blinded.  "Outcome assessors were blinded to treatment allocation." |
| Incomplete outcome data | Unclear risk | "Multiple imputations method was applied to the missing data for the primary outcome only." |
| Intention-to-treat analysis | Low risk | "Treatment evaluations were performed on the principle of intention to treat…." |
| Selective reporting | Low risk | One secondary outcome specified in trial registration (6-minute walk test) not reported. |
| Other sources of bias | Low risk | No other risks identified. |

**Maddison 2019 (45, 69)**

| Design | | Two arm parallel non-inferiority RCT + process evaluation | | | |
| --- | --- | --- | --- | --- | --- |
| Protocol/trial registration | | Yes/Yes (ACTRN12614000843651) | | | |
| Participants  Eligibility | | n=162 (intervention = 82, control = 80). Mean age=61±13.2 intervention, 61.5±12.2 control. 86% Male.  **Inclusion**  Clinically stable CHD patients, English speaking, ≥18 years with a diagnosed with CHD within 6 months (atherosclerosis, angina pectoris, myocardial infarction, coronary revascularization)  **Exclusion**  Hospital admission for CHD within 6 weeks, terminal cancer, pacemaker or implantable cardioverter-defibrillator, significant non-CHD exercise limitations; contraindicated for maximal exercise testing. | | | |
| Treatments | | **Intervention:** REMOTE-CR  ICT: bespoke REMOTE-CR telerehabilitation platform comprising smartphone, chest-worn wearable sensor (BioHarness 3, Zephyr Technology, USA), mobile and web apps.  Content: 12 weeks (36 sessions) of individualized real-time remote exercise prescription, monitoring, coaching and exercise behavior change support.  **Control:** Center-based exercise CR.  12 weeks (36 sessions) of individualized face-to-face exercise prescription and coaching. | | | |
| Outcomes | | **Primary:** Exercise capacity (VO_2_max (mL/kg/min) at 12 weeks  **Secondary:** Fasted blood lipid (total, high-density and low-density lipoprotein cholesterol; triglyceride) and glucose concentrations, anthropometry (height, weight, BMI), blood pressure (systolic/diastolic), PA, exercise-related motivation and adherence and health-related quality of life. Economic and process analyses.  **Implementation:** Acceptability, appropriateness, feasibility, fidelity, implementation cost. Level of analysis: participant/consumer. | | | |
| Follow-up | | 12 weeks and 24 weeks | | | |
| Country | | New Zealand | | | |
| Risk of bias | | Judgement | | Support for judgement | |
| Sequence generation | | Low risk | | "Participants were randomized (1:1)…using a computer-generated sequence—created by a blinded statistician—that included variable blocking (n=2/4) and stratification (sex/study site)." | |
| Allocation concealment  Blinding of participant/personnel | | Low risk  High risk | | "Treatment allocation was concealed until completion of baseline assessment in sequentially numbered, sealed, opaque envelopes."  No participant blinding. | |
| Blinding – outcome assessment | | High risk | | "…staff performing V̇O_2_max testing at 12 weeks were blinded to treatment allocation". Other outcomes appear unblinded | |
| Incomplete outcome data | | Unclear risk | | "Multiple imputations were applied to missing primary (but not secondary) outcome data using the Markov chain Monte Carlo method assuming the data were multivariate normally distributed and the missing data were missing at random." | |
| Intention-to-treat analysis | | Low risk | | "Treatment evaluations were performed on the principle of intention-to-treat." | |
| Selective reporting | | Low risk | | All pre-specified outcomes were reported. | |
| Other sources of bias | | Low risk | | No other risks identified | |

**Park 2014 (62)**

| Design | Two arm parallel RCT | |
| --- | --- | --- |
| Protocol/trial registration | No/No | |
| Participants | n=90 (intervention A=30, intervention B=30, control=30). Mean age= 59.2. 76% male.  **Inclusion**  Age ≥ 21 years, hospitalized for non-ST elevation MI, ST-elevation MI, or PCI, prescribed an antiplatelet medication, prescribed a statin medication, had a mobile phone with text messaging capability and able to speak, read and understand English.  **Exclusion**  Cognitive impairment and inability to operate a mobile phone | |
| Treatments | **Intervention**  ICT: CareSpeak Communications "mobile Health manager" platform (New Jersey) + mobile phone.  Content (Intervention A): Personalized, non-interactive SMS health education + medication reminders.  Content: (Intervention B): Personalized, non-interactive SMS, health education only  **Control**: No SMS | |
| Outcomes | **Primary:** Medication adherence among patients  **Secondary:** feasibility and patient satisfaction  **Implementation:** Acceptability, appropriateness, feasibility, fidelity. Level of analysis: participant/consumer. | |
| Follow-up | 30 days | |
| Country | United States of America | |
| Risk of bias | Judgement | Support for judgement |
| Sequence generation | Low risk | "Group assignment was generated by random allocation sequence using blocks of six that was prepared by a biostatistician". |
| Allocation concealment  Blinding of participant/personnel | Low risk  High risk | "The PI assigned patients to their groups by distributing [sealed opaque] envelopes in consecutive, numbered order".  "Due to the nature of the study design, the PI and patients could not be blinded to the intervention". |
| Blinding – outcome assessment | Unclear risk | Unclear who conducted outcome assessment. PI was not blinded to treatment allocation. |
| Incomplete outcome data | Low risk | Minimal loss to follow-up, balanced across groups. |
| Intention-to-treat analysis | Low risk | "All data were analyzed with intention to treat". |
| Selective reporting | Low risk | All specified outcomes were reported; a published protocol was not available to verify completeness. |
| Other sources of bias | Unclear risk | "Use of Medication Event Monitoring System (MEMS could have unintentional attention to regular medication-taking habits for all groups…” |

**Piotrowicz 2014 (63, 70)**

| Design | Uncontrolled pre-post intervention study | |  |
| --- | --- | --- | --- |
| Protocol/trial registration | No/No | |  |
| Participants  Eligibility | n=365, (333 Coronary artery disease patients). Mean age=58.3±10.5 years. 64% male.  **Inclusion**  “Left ventricular ejection fraction >40%, New York Heart Association class I or II, no evidence of congestive heart failure and myocardial ischemia or angina at rest or on the exercise test at or below 6 metabolic equivalents, ability to self-monitor intensity of activity and patients willingness to comply with the program”.  **Exclusion**  Unstable angina, congestive heart failure, uncontrolled hypertension, symptomatic and/or exercise-induced cardiac arrhythmia or conduction disturbances, congenital heart disease requiring surgical treatment, impaired renal or hepatic function, acute and/or decompensated non-cardiac disease and physical disability. | |  |
| Treatments | **Intervention:** Home-based cardiac telerehabilitation  ICT: Tele-electrocardiogram + blood pressure monitor (Pro Plus, Poland) + mobile phone.  Content: Two-stage intervention had two stages: Initial (3 days - baseline clinical examination + psychological assessment + educational sessions at outpatient center); Basic (4 weeks – Remotely monitored tele-electrocardiogram + blood pressure monitoring during 5 training sessions per week).  **Control:** No control group | |  |
| Outcomes | **Primary:** Feasibility  **Implementation:** Appropriateness, feasibility and fidelity. Level of analysis: participant/consumer. | |  |
| Follow-up | 4 weeks | |  |
| Country | Poland | |  |
| Risk of bias (Joanna Briggs Institute Critical Appraisal Checklist for Cohort Studies) (59) | | Judgement | |
| Were the two groups similar and recruited from the same population? | | Not applicable | |
| Were the exposures measured similarly to assign people to both exposed and unexposed groups | | Not applicable | |
| Was the exposure measured in a valid and reliable way? | | Yes | |
| Was confounding factors identified? | | No | |
| Were strategies to deal with confounding factors stated? | | No | |
| Were the groups/participants free of the outcomes at the start of the study (or at the moment of exposure)? | | Not applicable | |
| Were the outcomes measured in a valid and reliable way? | | Yes | |
| Was the follow up time reported and sufficient to be long enough for outcomes to occur? | | Yes | |
| Was follow up complete, and if not, were the reasons to loss to follow up described and explored? | | Yes | |
| Were strategies to address incomplete follow up utilized? | | Not applicable | |
| Was appropriate statistical analysis used? | | Yes | |

**Salvi 2018 (64, 71)**

| Design | Multi-country two arm parallel RCT + process evaluation | |
| --- | --- | --- |
| Protocol/trial registration | No/Yes (NCT01761448) | |
| Participants  Eligibility | n = 118, (intervention=55, control=63). Mean age=59 ± 14 years. 89% male.  **Inclusion**  CAD patients after acute MI/elective coronary intervention; ejection fraction >30%; willing to exercise; eligible for standard local rehabilitation; ability to use computer/Internet.  **Exclusion**  EF <30%; New York Heart Association class IV heart failure; unable to exercise; severe valve disease; cardiac surgery within 4 weeks; implanted cardiac devices (cardioverter-defibrillator, resynchronization, pacemaker); open thorax wound. | |
| Treatments | **Intervention:** Web and mobile guided exercise (GEx) system  ICT: mobile station (wearable sensor, smartphone + mobile app), patient station (tablet PC + software); professional station (website).  Content: Mobile station - monitoring physical exercise and providing live guidance, Patient station - collector and gateway of patient's data and delivering educational content, and Professional station - a web-based application used by doctors to prescribe and tailor each exercise programme.  **Control:** Standard rehabilitation as per national procedures in each country | |
| Outcomes | **Primary:** User acceptance and perceived usefulness, exercise adherence  **Implementation:** Acceptability, appropriateness, fidelity, feasibility. Level of analysis: participant/consumer. | |
| Follow-up | 6 months | |
| Country | Spain, Germany and the UK | |
| Risk of bias | Judgement | Support for judgement |
| Sequence generation | Low risk | Sequence generation method not described in detail but risk appears low. |
| Allocation concealment  Blinding of participant/personnel | Low risk  Unclear risk | "Randomization stratified by 'gender' and 'center' was done electronically by the system…”  Not described. |
| Blinding – outcome assessment | High risk | "…un-blinded design seemed to be appropriate to reach primary objectives." |
| Incomplete outcome data | High risk | High drop out in intervention, 36 (66%) and control group, 21 (33%). At 6 month follow up only 8/55 in intervention and 7/63 were analyzed). |
| Intention-to-treat analysis | High risk | Analysis appear to be per-protocol. |
| Selective reporting | High risk | No protocol available. |
| Other sources of bias | High risk | "…low number of participants and relatively high number of dropouts. This can potentially introduce bias in the perception of the system as only limited number of motivated patients completed the study”. |

**Varnfield 2014 (44)**

| Design | Two arm parallel RCT | |
| --- | --- | --- |
| Protocol/trial registration | No/Yes (ANZCTR12609000251224) | |
| Participants  Eligibility | n=120 (intervention=60, control=60). Mean age=56.2 ±10.1 (intervention), 54.9±9.6 (control). 83% male (intervention), 91% male (control).  **Inclusion**  MI; referred for CR.  **Exclusion**  Unable to participate in a self-management programme; no experience with or unable to operate a smartphone; unable to attend centre-based exCR; involved in another trial. | |
| Treatments | **Intervention:** Care Assessed Platform of CR (CAP-CR)  ICT: smartphone, mobile apps, website, telephone  Content: Web- and smartphone-based multifactorial CR program including monitoring health and exercise, delivering educational SMS, and preinstalled audio/video education about CVD symptoms and management. Interaction with health professionals via a web portal and telephone.  **Control:** center-based CR (2 supervised exercise and 1-hour educational sessions on a weekly basis for 6 months) | |
| Outcomes | **Primary:** Exercise uptake, adherence, and completion of follow-up.  **Secondary:** Blood pressure, blood lipid/glucose concentrations, body composition, physical activity, clinical events.  **Implementation:** Appropriateness, adoption, fidelity. Level of analysis: participant/consumer. | |
| Follow-up | Post-treatment (1.5 months) and long-term follow-up (6 months post-randomization). | |
| Country | Australia | |
| Risk of bias | Judgement | Support for judgement |
| Sequence generation | Low risk | *"Permuted block randomization, by computer-generated random numbers with variable block sizes..."* |
| Allocation concealment  Blinding of participant/personnel | Low risk  High risk | *"…using sequentially numbered opaque, sealed envelopes."*  Not blinded. |
| Blinding – outcome assessment | High risk | *"We conducted an unblinded RCT...""* |
| Incomplete outcome data | Unclear risk | Significant and variable missing data across outcomes. Mixed methods for handling missing data. |
| Intention-to-treat analysis | Unclear risk | As above, Intention To Treat (ITT) analysis for exercise uptake and completion. Per protocol analysis for remaining outcomes. |
| Selective reporting | High risk | Not all outcomes specified in methods/trial registration are reported—some outcomes not reported at all specified time points. |
| Other sources of bias | High risk | A priori primary outcome changed due to low recruitment. |

CAD, coronary artery disease. CAP-CR, care assessment platform. CHD, coronary heart disease. COPD, coronary obstructive pulmonary disease. CR, cardiac rehabilitation. CVD, cardiovascular disease. ITT, intention to treat. MET_peak_, peak metabolic equivalent of task. MI, myocardial infarction. RCT, randomized controlled trial. SMS, short message service.
